# Assessing MMA Welding Process Stability Using Machine Vision-Based Arc Features Tracking System

**DOI:** 10.3390/s21010084

**Published:** 2020-12-25

**Authors:** Wojciech Jamrozik, Jacek Górka

**Affiliations:** 1 Department of Fundamentals of Machinery Design, Silesian University of Technology, 44-100 Gliwice, Poland; 2 Department of Welding Engineering, Silesian University of Technology, 44-100 Gliwice, Poland; jacek.gorka@polsl.pl

**Keywords:** MMA welding, welding arc, vision system, process monitoring, welding arc stability

## Abstract

Arc length is a crucial parameter of the manual metal arc (MMA) welding process, as it influences the arc voltage and the resulting welded joint. In the MMA method, the process’ stability is mainly controlled by the skills of a welder. According to that, giving the feedback about the arc length as well as the welding speed to the welder is a valuable property at the stage of weld training and in the production of welded elements. The proposed solution is based on the application of relatively cheap Complementary Metal Oxide Semiconductor (CMOS) cameras to track the welding electrode tip and to estimate the geometrical properties of welding arc. All measured parameters are varying during welding. To validate the results of image processing, arc voltage was measured as a reference value describing in some part the process stability.

## 1. Introduction

**Figure 1:**
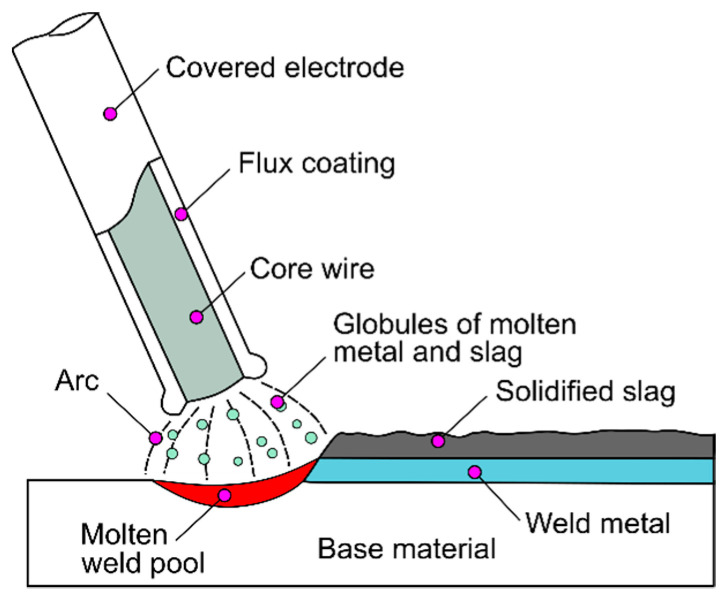
The idea of manual metal arc (MMA) welding.

Manual metal arc welding (MMA—manual metal arc—111) is a process of permanently joining metals by melting a filler material, called a binder, and the edges of the base material using a heat source (Figure 1). The coated electrode welding method is used to join practically all types of steel (unalloyed steels, high-strength steels, energy steels, high-alloy steels), copper, nickel, and even grey cast iron in repair welding [1,2]. The heat source in this method is an electric arc glowing between the core of the coated electrode and the surface of the welded material. The temperature of this arc is between 2400 and 5000 °C. The arc length should be constant and equal to the core diameter. Changes in the arc voltage cause instability of the welding process and the formation of welding imperfections. MMA arc welding can be conducted with direct current with negative DC(−) or positive DC(+) polarity and with alternating current. The welder, guiding the electrode along the joint, moves it in at least two directions, downwards, as the electrode fuses, towards the weld pool, keeping the arc length constant, and along the joint, filling the weld groove with filler [3,4]. Additionally, the welder can perform transverse, swinging movements with the tip of the electrode. This creates conditions in which it is very difficult to maintain a constant arc length and therefore a constant arc voltage. The arc voltage is proportional to the arc length and affects the nature of metal transfer in the arc, welding speed and the depositing efficiency of the weld metal. As the arc voltage increases, the arc energy and hence the volume of the weld pool increases, but especially the width and length of the weld pool. At a constant current intensity, the increase in the arc voltage slightly affects the penetration depth. When the arc is too long, the heat is intensively dissipated to the atmosphere and the metal flux transferred from the electrode to the weld pool is significantly splashed. DC current provides a more stable arc and even metal transfer throughout the arc, even at low currents. The edge fusion of the sheets is also higher and the arc shortening tendency is lower. The polarity of the direct current determines the nature of the metal transfer in the arc, the fusion rate of the electrode and the depth of fusion. A higher fusion rate is obtained with negative polarity, and a greater fusion depth with positive polarity. Certain grades of electrodes, e.g., low hydrogen basic electrodes, intended for welding austenitic steels and non-ferrous metals and high-strength steels, require very high stability of the arc glow and can only be fused with direct current with positive polarity. In the case of welding with electrodes with a basic coating with negative polarity, large spatter appears, and the arc is unstable. With positive polarity, the arc glows steadily. According to that, the wrong connection of wires, which can lead to polarity change, is highly unwanted and should be avoided by welders. In particular, unexperienced welders are vulnerable to making such mistakes [5,6,7].

**Figure 2:**
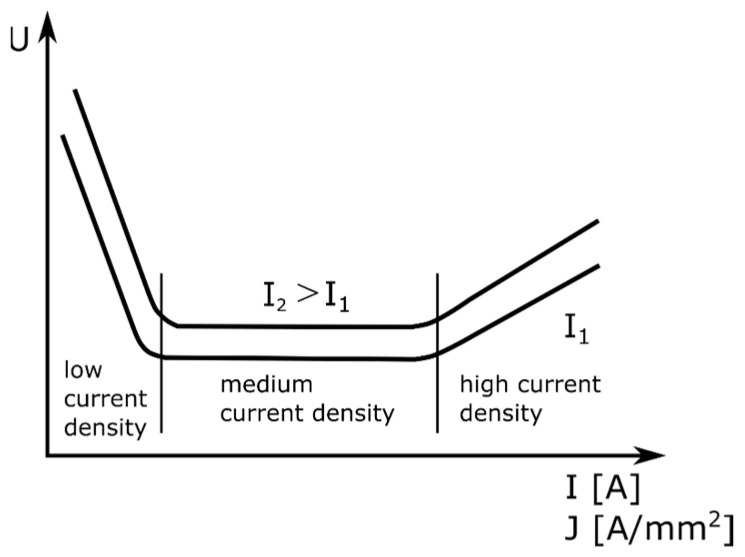
Static characteristics of an electric arc.

An electric arc is an electric discharge in an atmosphere of highly ionized gases and metal vapors. The necessary condition for the arc to glow is ionization of the gas filling the space between the electrodes, i.e., there must be electric charges (positive ions, negative ions, and electrons). An electric arc can arise as a result of a flashover between the electrodes that are spaced a short distance apart or the contact of the electrodes followed by their separation. In the first case, ionizers generating high-frequency pulses with a voltage of several thousand volts (e.g., tungsten inert gas (TIG) welding) are needed to cause the flow of electrons. In the second, commonly used case, a voltage of several dozen volts is enough (e.g., welding with coated electrodes or a submerged arc). The electric arc widens conically towards the surface to be welded, regardless of the polarity of the welding current. Three zones are distinguished in the ionized space between the electrodes: the cathodic voltage drop space (U_C_), the arc pole (U_AR_), and the anodic voltage drop space (U_A_). By measuring the current J and the voltage U while the arc is glowing, it is possible to determine the static characteristics of the arc (Figure 2). The static characteristic of the arc shows the relationship between the voltage and the current of the arc: U = U_C_ + U_A_ + U_AR_ = f (j).

**Figure 3:**
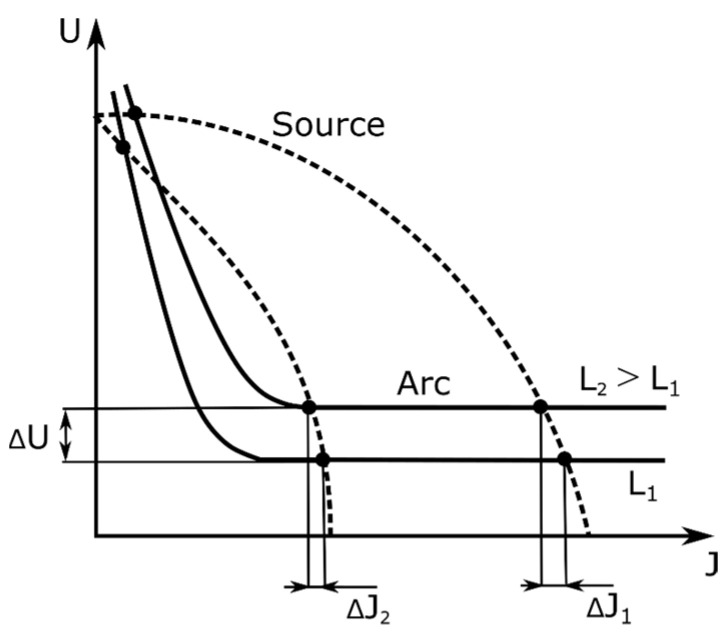
Changes in parameters in the case of welding with average current densities.

In welding, the flat and rising static characteristics of the arc are practically used. Due to the characteristics of the arc, the power sources intended for welding must have special properties. In the case of welding with coated electrodes, the power source must have a steep characteristic. When welding with coated electrodes, it is desirable to maintain a constant welding current with the inevitable changes in the arc length and the related changes in the arc voltage. To allow the arc to be struck and maintained, the voltage at the terminals of the current source must vary with the intensity of the current in such a way that the static characteristics of the current source intersect the static characteristics of the arc at two points (Figure 3).

Welding arcs glow brightly, and that glow comes from the radiation that the arc produces. Part of the radiation is visible light, in the 400 to 700 nm range, while the other part of the radiation is infrared (700 to 1400 nm) and is the part that produces heat. There is also some UV radiation in the range of 200 to 400 nm.

Monitoring of MMA is a crucial task, as the quality is highly dependent on the skills of a welder who must maintain the arc length and the welding speed at a constant level. During the arc welding process, there are various kinds of information, such as electric arc voltage, welding current, welding sound, weld pool shape, etc., which are closely related to the welding quality. A skilled welder can generally evaluate the condition of the seam, judging several signals based on the experience. Considering the highly nonlinear and time-varying complex welding process with a high number of degrees of freedom MMA is not often a subject of research. Several methods utilizing one or more sources of information about the welding process condition and stability was elaborated for different welding techniques [8,9,10].

In [11,12,13], a technique of modelling the welder response to welding pool surface was presented. The welding current as a response to the 3D weld pool surface, as characterized by its width, length, and convexity, was studied, and human welder behavior modelled. A method of welding skill evaluation using high-speed welding current and arc voltage in MMA was also elaborated [14,15]. In this method, the probability density distributions was calculated based on recorded electrical signals and the differentiation between experienced and novice welders was made. Other sensing and reasoning techniques are also proposed to limit or reduce the appearance of defects like the arc voltage sensor [16,17,18], acoustic emission measurements [19], thermography [20,21], vision light cameras [22] and sound [23,24]. Additionally, there are many multisensory techniques that are often supported by artificial intelligence, showing in some cases of welding monitoring an impressive performance regarding accuracy and robustness. As an example, the multisensor information fusion technology was applied to predict the penetration status in pulsed TIG [22,23,24,25]. In [26], the depth and width estimation of the weld bead in TIG based on multisensor information fusion and an artificial neural network was proposed. Most studies presented results obtained on mechanized and robotized stands for welding methods that are carried out in an automated manner, such as gas metal arc (GMA) and TIG welding. This is the case for arc length–arc voltage relationship investigation [27,28,29,30] and for the monitoring of the welding process using vision systems [31,32,33].

Monitoring of the welding arc [34] and the weld bead [35,36] is a common and vital scientific task. There are several types of features, that can be used to assess the process stability or joint quality: statistical features, describing the luminance (in certain wavelength) or temperature distribution in the welding arc area, geometrical features, reflecting the shape of the welding arc and profile-based features, where for temperature profiles that cross the welding arc, any disturbance in the profile symmetry can indicate the appearance of the instability of the welding process [34]. In many approaches, the key issue is the correct image binarization, in order to determine the geometrical and topological features of observed objects and regions [37,38]. In the case of machine vision systems that are based on visible light cameras, the application of statistical and profile-based features is debatable, because when taking images in the whole wavelength range offered by the camera, the results are dependent on the camera matrix type, aperture adjustment and protective glass or filter properties. Geometrical features and some dependencies among them are more robust in the context of camera setup changes. Moreover, the application of profile-based features demands tracking of characteristic points in the welding arc area connected with the electrode or welding wire tip position. Any inaccuracy and deviation in profile placement will lead to incomparable results.

In the paper, the method for evaluating the MMA welding process stability and polarity using CMOS cameras, aiming to make the MMA welding training and controlling the stability of the welding process easier, is presented. The aim of the performed research was to find appropriate image processing and analysis methods, as well as the implementation of elaborated methods on a cost-optimized hardware setup. To obtain generalization of the method suitability for MMA welding stability and polarity assessment, different welding parameters were used to generate test samples on which the proposed method was adjusted and verified. The rest of the paper is organized as follows. In Section 2, the used specimen material, welding device and welding consumables are listed. The measurement system is described and signal (thermograms and visible light images) processing routines are characterized. Additionally, the welding experiment is explained. In Section 3, there is description of the obtained results. The analysis and assessment of welding arc stability and the detection of current polarity is gathered in this section. The formulas used to assess welding process condition are also included. End-user hardware realization of the system that applies the elaborated method is mentioned in Section 5. The entire research is finally concluded in Section 5.

## 2. Materials and Methods

All tests were performed with an inverter source ESAB CADDY TIG 1500i TA34, ESAB AB., Göteborg. Sweden. Stick electrodes Lincoln Electric BASIC 7018, Bielawa, Poland (size 3.2 × 350 mm) were chosen. The chemical composition and mechanical properties of used electrode type are in Table 1. The manufacturer suggests applying 100–140 A DC(+) current when using this type of elector. Basic coated electrodes are intended for welding low-alloy structural steels with increased and high-strength properties. The basic coating provides the weld with high metallurgical purity as well as high strength and plastic properties. Only electrodes with an alkaline outline are characterized by the low-hydrogen welding process, which reduces the risk of cold cracks. These are the electrodes most sensitive to changes in the arc length; its elongation disturbs the process stability and, consequently, leads to welding defects.

Test specimens were made from S355J2, a weldable high-strength alloy steel. Chemical composition and properties of used materials are gathered in Table 2. The metal plate from which samples were prepared was 12 mm thick.

The arc voltage was measured using a NI USB-6009, NI, Austin, TX, USA card with acquisition rate of 25 kHz. Before feeding the measuring transducer, the voltage was conditioned using LEM LV 25P voltage transducer to a voltage range 0–10 V, that was suitable for acquiring. Sequences of the welding arc were recorded using a visible light camera, as well as an infrared camera (thermographic camera). A Dalsa Genie Nano C2050, Teledyne DALSA, Waterloo, Canada with a CMOS matrix and 25 mm lens was used to as the visible light camera (VIS CAM). The recording was performed with a rate of 30 fps, and the frame size was 2064 × 1544 pixels with the 8-bit colour depth. To protect the camera sensor against blinding by the welding arc a protective glass with shading grade 12 was applied. No additional filters were applied. Thermograms were acquired with an infrared FLIR A655sc camera, FLIR Systems, Wilsonville, USA (Infrared Radiation (IR) CAM, spectral range 7.5–14.0 µm) with a 25 mm lens. Thermograms were taken with 200 fps and the spatial resolution was 640 × 120 pixels. Because there was no need to know the exact temperature in the arc or the welding pool, a constant emissivity ε = 0.95 was used. This assumption allowed further comparison of geometrical and topological properties of welding arc in different welding process conditions. Optical axes of both cameras were perpendicular to the surface of the metal plate used for cladding. Measuring devices were triggered to start acquisition simultaneously. The acquisition software was written in NI LabVIEW 2017. For the IR camera, a dedicated software FLIR ResearchIR x64 was used. The PC used as the system central unit was equipped with: Intel Core i7-7700K, 4.2 GHz, ASUS TUF Z270 MARK 2 motherboard, Corsair Vengeance LPX 16 GB DDR4 3000 MHz RAM.

**Figure 4:**
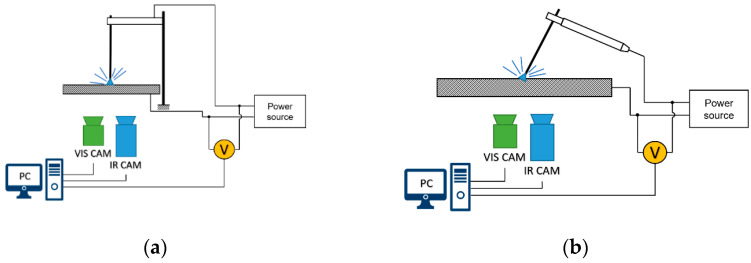
Illustration of test stands used in research: (**a**) stand for welding arc examination; (**b**) manual cladding process.

Two test scenarios were taken into consideration to assess the behavior of the welding arc in different conditions. In the first scenario, a test stand for the examination of the maximal welding arc length were used (Figure 4a). In this case, the welding rod was fixed on a constant height, with a gap between the welding rod tip and the welded plate of about 2 mm. After the ignition of the welding arc using an external metal plate to induce the contact between the electrode and the welded metal, the welding arc glows until its length exceeds a critical value that causes the breaking of the arc. In this case, DC(+) and DC(−) polarities were used and the welding current was set to 110, 130 and 150 A DC. For the used rod, the optimal welding current should be 130–140 A DC, and the arc length should be 4.0 mm. For each welding current and polarity, three attempts were made. Finally, 18 trials were made and acquired.

The second procedure was an MMA cladding (Figure 4b). In this case, the welding current was set to 140 A DC and both DC(+) and DC(−) polarities were applied. Cladding was performed in three different ways:

- Stable—short arc. The welder kept a constant arc length, which was optimal for the selected welding stick.
- Unstable—long arc. The welder tried to conduct cladding with the longest possible arc. If the air gap becomes too large for the voltage, the arc may be extinguished during this procedure.
- Mixed stability. The welder changed the arc length during the procedure.

All data acquired during the research were processed in order to create signals that can be used to assess process stability in terms of welding arc length and change dynamics what was connected to the used polarity. The main issue was to elaborate a processing and analysis method for the VIS images that will allow the evaluation of welding process properties during welding (cladding). All processing and analysis methods were elaborated in a MATLAB 2020b environment.

Arc voltage and thermogram sequences were used as benchmark data sources, that can be used to confirm the usefulness of features that were extracted from VIS image sequences. Voltage signals were filtered with a low pass filter with the passband frequency of 5 kHz. A sliding window was applied to the filtered signal to calculate voltage RMS. The width of the window was 100 samples.

**Figure 5:**
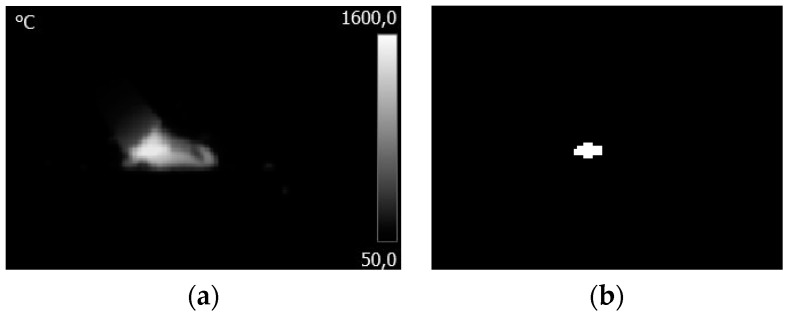
Processing steps of IR CAM images: (**a**) original image; (**b**) binarized and cropped image.

To assess infrared images, a set of geometrical features were extracted for each frame. Before the feature extraction stage, each thermogram was thresholder to obtain a binary image (Figure 5). Thermograms were binarized with a fixed threshold, that was the same for all sequences tr_IR_ = 1890 °C (apparent). The temperature value was selected as a result of the searching algorithm. The searching algorithm was elaborated to find a threshold that will produce binary images with the largest continuous binary object that would represent the welding arc and the elimination of the hot seam that is also visible in the IR image at the same moment. The boundary conditions for the threshold were set to 1500–2000 °C. For the binary IR images with one object that can be regarded as a region of interest (ROI), basic geometrical and topological properties were calculated. The set of features contained centroids (coordinates of center of mass of the region), lengths of the major and minor axes of the ellipse that has the same normalized second central moments as the ROI, perimeter and area of ROI. Centroids were used to evaluate the stability of the process, taking into consideration welder skills such as ability to keep a constant arc length and constant travel (welding) speed. Other geometrical features were used to assess the arc length and polarity in a way that would be independent of the configuration of measuring equipment, especially absolute values of temperature and distance between the IR camera and stick electrode. To minimize the influence of high frequency disturbances that were present in the features, signals peak envelopes were calculated and then analyzed. Pixel sizes on the thermogram were converted to real life units. Taking into consideration camera configuration, PS = 17μm and distance to target WD = 500 mm (Working Distance), horizontal field-of-view (HFOV) = 220 mm, and VFOV 170 mm (Vertical Field-Of-View), as well as IFOV = 0.35 mm (Instantaneous Field-Of-View, the size of smallest recognizable object).

**Figure 6:**
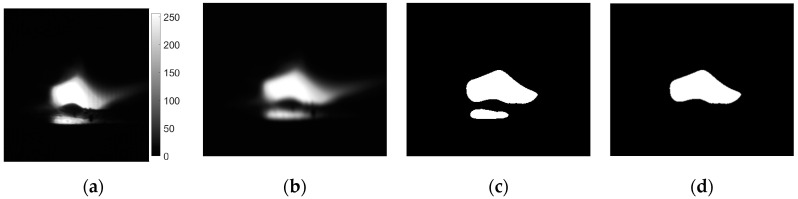
Processing steps of visible light camera (VIS CAM) images: (**a**) original image—arc luminance; (**b**) low-pass filtered image—arc luminance; (**c**) binarized image; (**d**) binarized image with only the arc region of interest (ROI) remaining.

Sequences taken by the VIS camera were first processed in order to remove artefacts which were the result of Bayer filtering (Figure 6a). Because there were no filters used, a full band of camera acquisition was used (about 400 to 950 nm). Because the brightness of the arc is connected with the amount of heat in high temperature iron vapor, which has a strong peak emission line at a 516.74 nm wavelength [39], and to also consider the outer cone of the electronic arc, that has a spectrum above 800 nm, and to reduce the influence of radiation that is close to UV (<500 nm), only the green and red camera channels were used. A low-pass filter was employed to remove high-frequency noisy pattern from the images. The next step was image thresholding (Figure 6b) to obtain binary (BW) arc representation. In the case of VIS images, the threshold was chosen arbitrarily on a level tr_VIS_ = 0.4 to keep high continuity of regions and to avoid reflexes that can lead to the multiplication of extracted ROIs. To overcome the situation when more than one objects were present in the binary image (Figure 6c), the larger object (with higher area) was always chosen for further analysis (Figure 6d). This was assumed as a sufficient representation of welding arc geometrical properties, because the proposed approach is not aimed at the exact measurement of welding arc length and orientation, but at assessing the welding process realization in a short time window based on the properties of the BW region containing representation of the welding arc in a broad wavelength range.

To ease the results’ implementation, VIS camera calibration was made in order to recalculate pixel size-based images to the real-life units. Taking into consideration camera parameters (resolution, pixel size on the camera matrix PS = 3.45 µm, focal length of the lens FL = 25 mm) and the working distance—the distance from the front of the lens to the electrode tip, WB = 500 mm—the field of view (FOV) and the pixel size in millimeters were calculated. The obtained results were: HFOV = 141.31 mm, VFOV = 105.98 mm, and IFOV = 0.069 mm.

## 3. Results and Discussion

### 3.1. Influence of Welding Current on Electric Arc Behaviour

**Figure 7:**
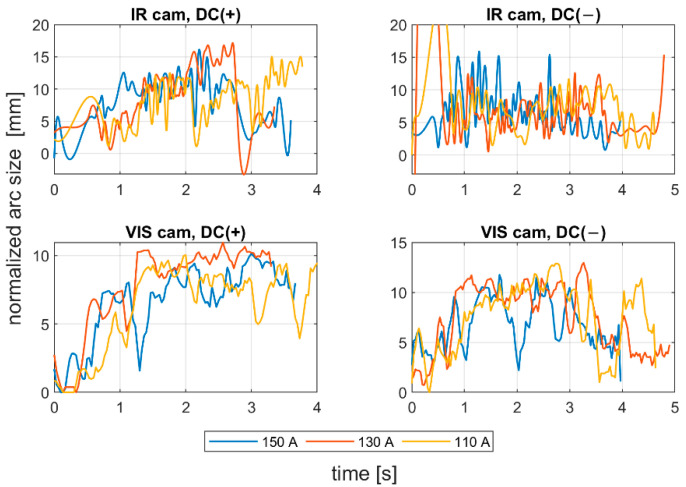
Comparison of normalized arc size, being the squared root of the minor and major axis length product of the ellipse, having the same second moment as the welding arc ROI for positive and negative polarity. For VIS CAM, the signal was averaged; for IR CAM, the signal peak envelope was calculated.

Image sequences obtained on the test stand for arc length measuring (Figure 4a) were subjected to a processing procedure described in Section 2. In Figure 7, there are plots of the normalized arc size, being the squared root of the minor and major axis length product. Those axes were calculated for the ellipse, having the same second moment as the welding arc ROI. Signals from VIS CAM were smoothed using the moving average method, while to assess changes in the IR CAM, an envelope signal was calculated. It can be easily seen that for the positive polarity, despite the welding current, all signals have an increasing characteristic, especially in the first part of the process, when the burning of the electrode was stable. It can be stated that the welding arc length is quite well-represented in the considered welding arc ROI feature, when dealing with VIS CAM images. In the case of the IR sequence, there are more disturbances in the signal. Nevertheless, the growing trend is also clear for the DC(+) polarity. For the negative polarity, especially in the IR CAM signal, there is a noticeable lack of process stability. There is no expected growth in the major axis length of the considered arc ROI. Taking into consideration weld current, for the stable DC(+) realizations, for the lowest current value, the process lasts for the longest time, but there are noticeable disturbances in the part, when the welding arc was long, and the air gap between electrode tip and the welded sample was still increasing. The process that was realized with the current set to 130A was the most stable.

The cross-correlation coefficients calculated between the diagnostic signal $z_{ARI}$ and corresponding voltage vectors were in a moderate range for the DC(+):0.60 for the unstable long arc and 0.44 for the stable, short arc length. After additional smoothing filtration, correlation factors increased to 0.72 and 0.63, respectively, for unstable and stable process realizations.

### 3.2. Evaluation of Arc Length Corectness

**Figure 8:**
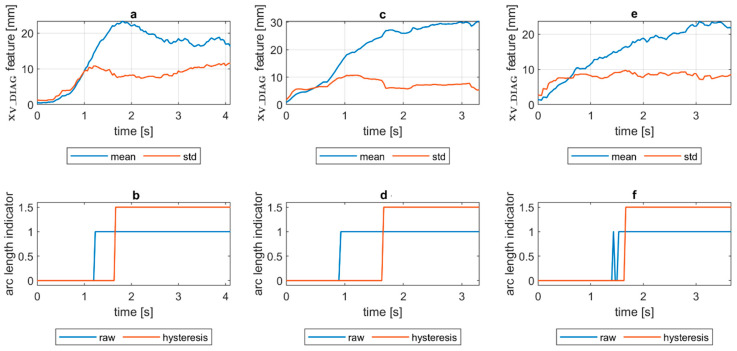
Change in arc length for DC(+) polarity on the test stand and the arc length indicator $z_{ALI}$ for welding current: (**a**,**b**) 110A; (**c**,**d**) 130A; (**e**,**f**) 150A.

To assess the welding arc length and automatically distinguish the length into two classes—short (correct) and long (incorrect)—a more robust feature was calculated. The diagnostic signal that will be the base for further analysis was constructed in the following manner:

(1) $$x_{V\_DIAG}=x_{V\_MIA}\cdot x_{V\_ECC}$$

where $x_{V\_MIA}$ is the minor axis length of the ellipse feature signal and $x_{V\_ECC}$ is a signal of the eccentricity of the ellipse that has the same second moments as the considered ROI. The ellipse eccentricity can be understood as the ellipse elongation, while it is the ratio of the distance between the foci of the ellipse and its major axis length (0 for circle, 1 for line). The $x_{V\_DIAG}$ underwent a moving average smoothing (${\bar{x}}_{V\_DIAG}$ called *mean*). Additionally, a moving standard deviation ($\sigma_{V\_DIAG}$ called *std*) was calculated for the diagnostic signal. In both cases, the length of moving window was 30 samples. In Figure 8, there is a comparison of ${\bar{x}}_{V\_DIAG}$ and $\sigma_{V\_DIAG}$ signals for DC(+) tests taken with different values of the welding current. It was found that with the increase in arc length, the span between the mean and standard deviation signals increases. Moreover, while the mean increases, the standard deviation remains on the same level. This phenomenon was the basis for the elaboration of a simple welding arc length indicator ($z_{ALI}$). The indicator takes the span between the ${\bar{x}}_{V\_DIAG}$ and $\sigma_{V\_DIAG}$ signals. The threshold value, which is used to distinguish between short and long arc welding, was given by a formula:

(2) $$z_{ALI}=\{\begin{array}{c} 1\;\mathrm{when}\;{\bar{x}}_{V\_DIAG}>A\;\mathrm{and}\;\sigma_{V\_DIAG}>B\\ 0\;\mathrm{otherwise} \end{array}$$

where $z_{ALI}$ = 1 for the long welding arc. The decision constants A and B were found using a genetic algorithm. Finally, A = 15 mm and B = 5.2 mm, but it has to be assumed that these values are only valid for the layout of the used test stand. The camera detector resolution, lens focal length and the distance from the camera to the welding rod are especially important to maintain. The binary arc length condition signal $z_{ALI}$ can raise the alarm in two modes. In the first, the raw values of the $z_{ALI}$ were used to trigger the fire alarm when a maximal acceptable level is exceeded. In the second mode, a response delay was set. In this mode, the arc overlength indicator was activated after the limit value was consecutively exceeded for the configured number of response delays (Figure 8).

### 3.3. Evaluation of Polarity Type

**Figure 9:**
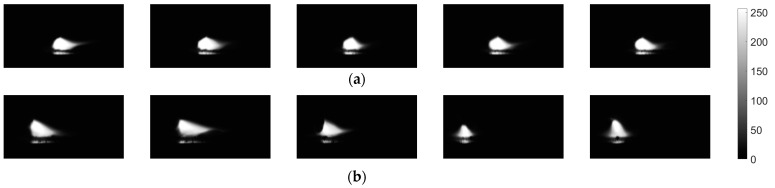
Five consecutive frames representing welding arc luminance taken by the VIS CAM: (**a**) short arc DC(+) cladding; (**b**) short arc DC(−) cladding.

Influence of polarity on arc shape is also visible when comparing several consecutive VIS frames acquired for the same electrode position with a horizontal plane (Figure 9). For the positive polarity, all images look very similar in terms of shape dimensions and luminance distribution in the arc area. For negative polarity, there are considerable changes in how the arcs behave. The flame moves around the welding tip in all directions. The arc cone changes its dimensions, so all geometry-based features vary, including area, perimeter, extent, etc.

Assessment of polarity was made in a different way than in the case of arc length. The metrics used were chosen to be independent of the absolute values of the thresholds. The same diagnostic signal $x_{V\_DIAG}$ was used in this case, but the averaging and standard deviation calculations were made in a short window, Δn=10 samples. The polarity indicator is defined as follows:

(3) $$z_{PLI}=\{\begin{array}{c} 1\;\mathrm{when}\;{\bar{x}}_{V\_DIAG\_2}-\sigma_{V\_DIAG2}>3\sigma_{V\_DIAG2}\\ 0\;\mathrm{otherwise} \end{array}$$

where $z_{PLI}$ = 1 for the negative polarity. Exceeding the limit may be also signaled in two ways: immediately after crossing the limit value and in the second approach after the signal exceeds the limit for certain number of response delays.

### 3.4. Arc Length-and Polarity Estimation in Real Process

**Figure 10:**
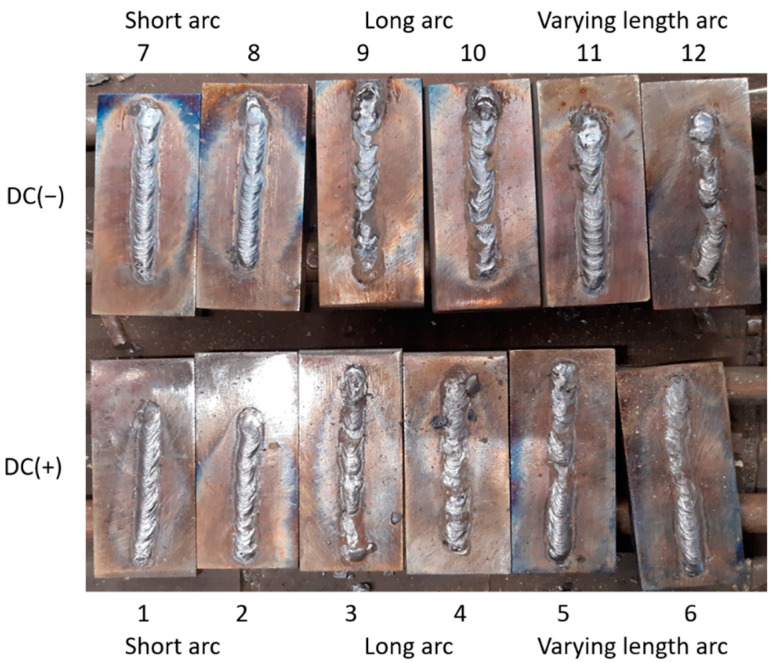
View of test clads made during the experiment with the constant welding current 140 A. In the top row, cladding was performed using DC(−) polarity, while for the samples in the bottom row, DC(+) polarity was applied. The welder performing the experiment made welds using three arc lengths: samples 1,2,7,8—a short, stable arc was used, and arc length was about 3.5 mm; samples 3,4,9,10—too long an arc was applied, and arc length was longer than 6mm; samples 5,6,11,12—the welder changed the arc length during the process, it has changed from short, stable arc, to long, unstable one.

Visual evaluation of welds revealed typical faults that are provoked by the arc length. When the arc is too long—in other words, when there is too great a distance between the rod and the work—the voltage will increase. This results in a flat and wide weld with a great deal of spatter. As the arc is quite unstable, there will be a real problem to remove risen slag from the edges of the weld. Too short an arc (at the begin of sample 11, Figure 10) produces a weld face that is uneven where it has been dragged along by the rod. For the short arc, the weld has a consistent profile, and the spatter rate is minimal.

**Figure 11:**
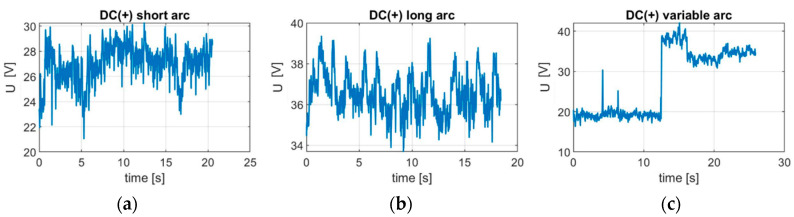
Voltage change caused by arc length variations for the DC(+) polarity: (**a**) stable—short arc (s2); (**b**) unstable—long arc (s3); (**c**) varying length arc (s5).

During the welding process it was noticed on the inverter welding machine screen that for the 140 A DC current, the voltage varied in the range 24–25 V when there was a correct and stable arc length. Measurements revealed (Figure 11) that this was the value for the optimal arc length. When the arc was long, the voltage was as high as 35–40 V. On the contrary, when the arc was too short, the voltage was as low as 20 V. This shows that the voltage change caused by welding arc length variations can exceed 20 V.

**Figure 12:**
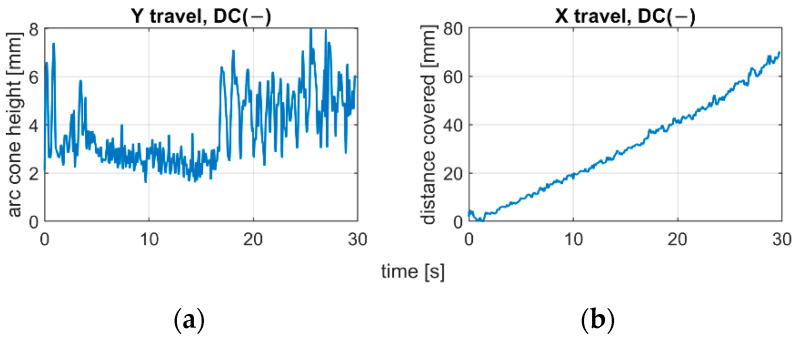
Centroid changes of welding arc ROI in IR image during negative polarity cladding with varying arc length (s12): (**a**) welding arc length estimate; (**b**) welding speed estimate.

**Figure 13:**
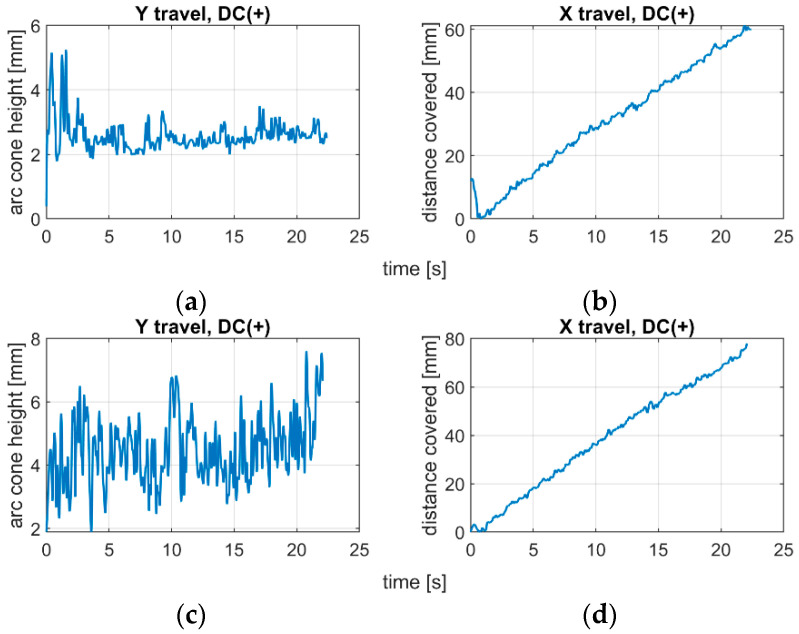
Centroid changes of welding arc ROI in IR image during positive polarity cladding with a constant arc length: (**a**) welding arc length estimate for short arc (s5); (**b**) welding speed estimate for short arc; (**c**) welding arc length estimate for long arc (s3); (**d**) welding speed estimate for long arc.

The applied polarity substantially influences the arc length and dynamics of arc change. Analyzing the position of centroids of arc ROI in the IR camera sequences, it was found that the negative polarity is characterized with fast changes in arc length, and due to highly unstable process realization, there is a difficulty in keeping the welding speed constant (Figure 12). For the positive polarity, when the arc is too long, the dynamics of arc geometry changes are also high, but the travel speed is easier to maintain on the same level during the whole process (Figure 13). The constant welding speed would be represented in the X travel path as a straight line. Adding a trend line to all X travel scenarios, the R^2^ for the positive polarity was around R^2^ = 0.98, while for negative polarity R^2^ < 0.93.

**Figure 14:**
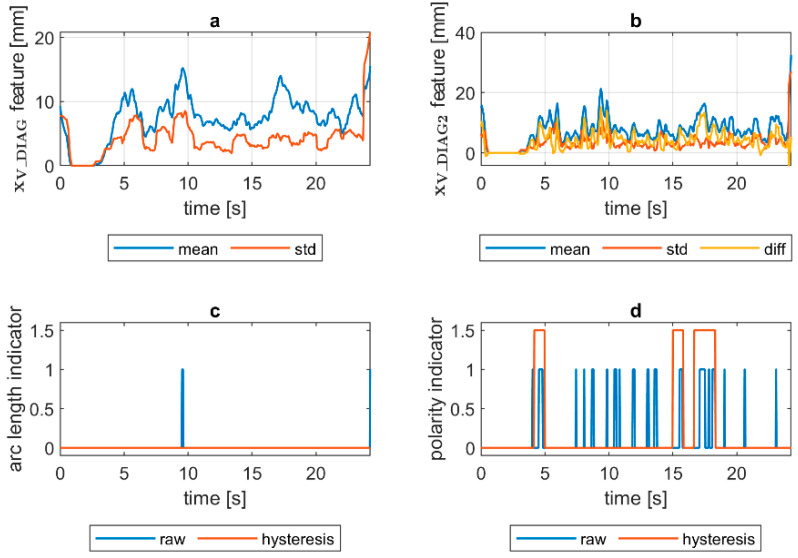
VIS CAM arc length ROI features for DC(+) short arc length process: (**a**) Sliding window mean ${\bar{x}}_{V\_DIAG}$ and sliding windows standard deviation $\sigma_{V\_DIAG}$ calculated for window length Δn=30 samples; (**b**) sliding window mean ${\bar{x}}_{V\_DIAG2}$ and sliding windows standard deviation $\sigma_{V\_DIAG}$ calculated for window length ∆*n* = 10 samples and difference among them ${\bar{x}}_{V\_DIAG}-\sigma_{V\_DIAG}$; (**c**) Welding arc length indicator ($z_{ALI}$) as a raw value and with a hysteresis lasting 30 response delays (samples); (**d**) Polarity indicator ($z_{PLI}$) as a raw value and with a hysteresis lasting 30 response delays (samples).

**Figure 15:**
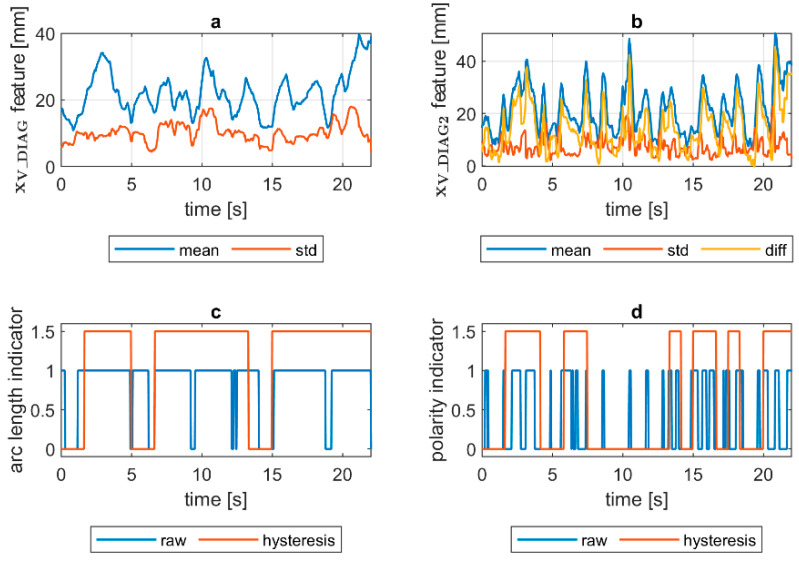
VIS CAM arc length ROI features for DC(+) long arc length process: (**a**) Sliding window mean ${\bar{x}}_{V\_DIAG}$ and sliding windows standard deviation $\sigma_{V\_DIAG}$ calculated for window length Δn=30 samples; (**b**) sliding window mean ${\bar{x}}_{V\_DIAG2}$ and sliding windows standard deviation $\sigma_{V\_DIAG}$ calculated for window length ∆*n* = 10 samples and difference among them ${\bar{x}}_{V\_DIAG}-\sigma_{V\_DIAG}$; (**c**) Welding arc length indicator ($z_{ALI}$) as a raw value and with a hysteresis lasting 30 response delays (samples); (**d**) Polarity indicator ($z_{PLI}$) as a raw value and with a hysteresis lasting 30 response delays (samples).

**Figure 16:**
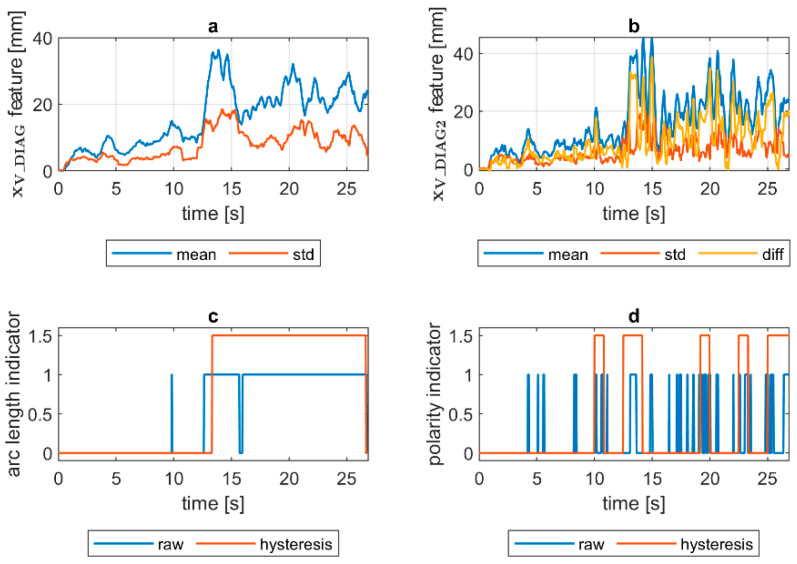
VIS CAM arc length ROI features for DC(+) varying arc length process: (**a**) Sliding window mean ${\bar{x}}_{V\_DIAG}$ and sliding windows standard deviation $\sigma_{V\_DIAG}$ calculated for window length Δn=30 samples; (**b**) sliding window mean ${\bar{x}}_{V\_DIAG2}$ and sliding windows standard deviation $\sigma_{V\_DIAG}$ calculated for window length ∆*n* = 10 samples and difference among them ${\bar{x}}_{V\_DIAG}-\sigma_{V\_DIAG}$; (**c**) Welding arc length indicator ($z_{ALI}$) as a raw value and with a hysteresis lasting 30 response delays (samples); (**d**) Polarity indicator ($z_{PLI}$) as a raw value and with a hysteresis lasting 30 response delays (samples).

**Figure 17:**
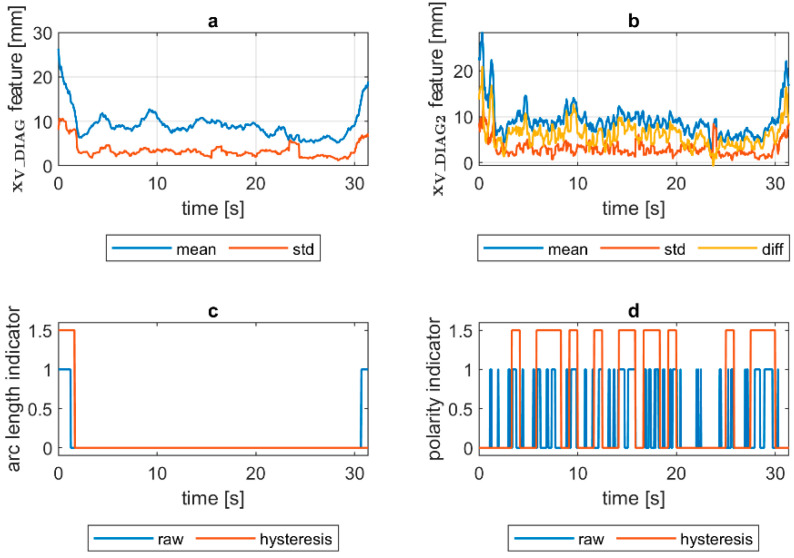
VIS CAM arc length ROI features for DC(−) short arc length process: (**a**) Sliding window mean ${\bar{x}}_{V\_DIAG}$ and sliding windows standard deviation $\sigma_{V\_DIAG}$ calculated for window length Δn=30 samples; (**b**) sliding window mean ${\bar{x}}_{V\_DIAG2}$ and sliding windows standard deviation $\sigma_{V\_DIAG}$ calculated for window length ∆*n* = 10 samples and difference among them ${\bar{x}}_{V\_DIAG}-\sigma_{V\_DIAG}$; (**c**) Welding arc length indicator ($z_{ALI}$) as a raw value and with a hysteresis lasting 30 response delays (samples); (**d**) Polarity indicator ($z_{PLI}$) as a raw value and with a hysteresis lasting 30 response delays (samples).

**Figure 18:**
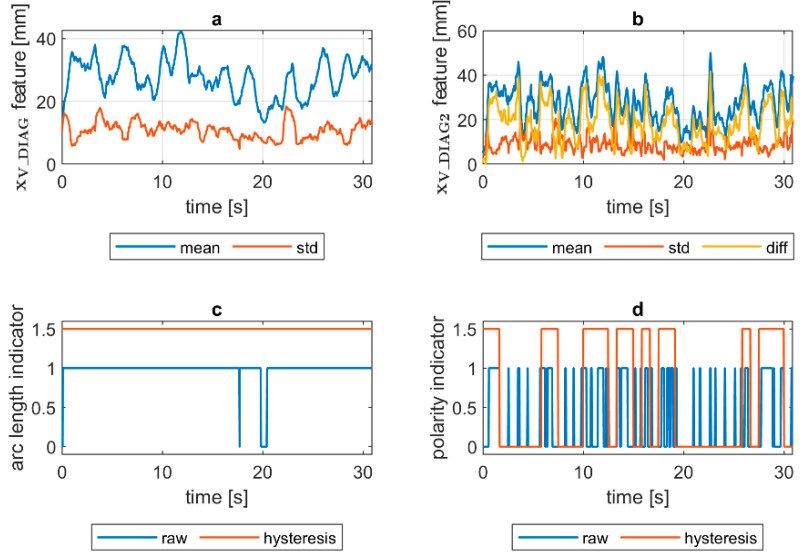
VIS CAM arc length ROI features for DC(−) long arc length process: (**a**) Sliding window mean ${\bar{x}}_{V\_DIAG}$ and sliding windows standard deviation $\sigma_{V\_DIAG}$ calculated for window length Δn=30 samples; (**b**) sliding window mean ${\bar{x}}_{V\_DIAG2}$ and sliding windows standard deviation $\sigma_{V\_DIAG}$ calculated for window length ∆*n* = 10 samples and difference among them ${\bar{x}}_{V\_DIAG}-\sigma_{V\_DIAG}$; (**c**) Welding arc length indicator ($z_{ALI}$) as a raw value and with a hysteresis lasting 30 response delays (samples); (**d**) Polarity indicator ($z_{PLI}$) as a raw value and with a hysteresis lasting 30 response delays (samples).

**Figure 19:**
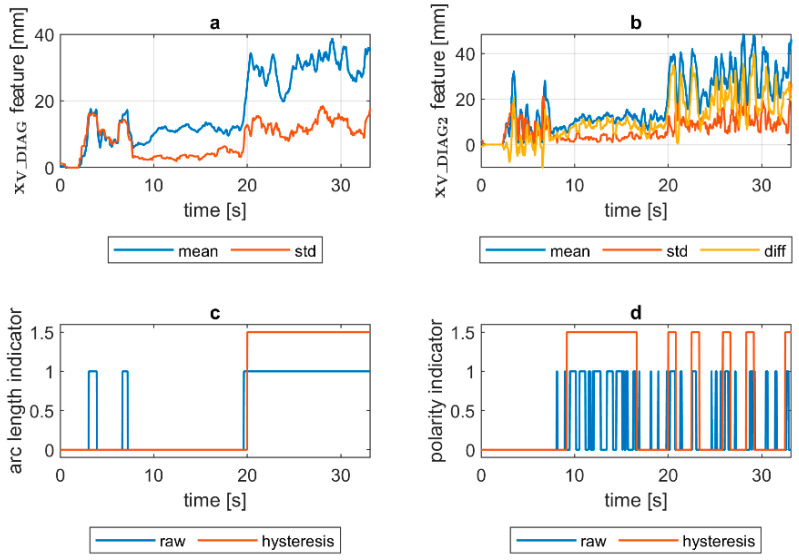
VIS CAM arc length ROI features for DC(−) varying arc length process: (**a**) Sliding window mean ${\bar{x}}_{V\_DIAG}$ and sliding windows standard deviation $\sigma_{V\_DIAG}$ calculated for window length Δn=30 samples; (**b**) sliding window mean ${\bar{x}}_{V\_DIAG2}$ and sliding windows standard deviation $\sigma_{V\_DIAG}$ calculated for window length ∆*n* = 10 samples and difference among them ${\bar{x}}_{V\_DIAG}-\sigma_{V\_DIAG}$; (**c**) Welding arc length indicator ($z_{ALI}$) as a raw value and with a hysteresis lasting 30 response delays (samples); (**d**) Polarity indicator ($z_{PLI}$) as a raw value and with a hysteresis lasting 30 response delays (samples).

Quantification of the welding arc length and polarity type was performed on all available samples. In Figure 14, Figure 15, Figure 16, Figure 17, Figure 18 and Figure 19, exemplary results are presented. In each figure, there are:

- Sliding window mean ${\bar{x}}_{V\_DIAG}$ and sliding windows standard deviation $\sigma_{V\_DIAG}$ calculated for window length Δn=30 samples.
- Sliding window mean ${\bar{x}}_{V\_DIAG2}$ and sliding windows standard deviation $\sigma_{V\_DIAG2}$ calculated for window length Δn=10 samples and difference among them $\left({\bar{x}}_{V\_DIAG2}-\sigma_{V\_DIAG2}\right)$.
- Welding arc length indicator ($z_{ALI}$) as a raw value and with a hysteresis lasting 30 response delays (samples).
- Polarity indicator ($z_{PLI}$) as a raw value and with a hysteresis lasting 30 response delays (samples).

Comparing means and standard deviations of the diagnostic signal in the longer time window (Figure 14, Figure 15, Figure 16, Figure 17, Figure 18 and Figure 19a), the mean feature value is always strongly connected with the arc length. It can be seen in Figure 16a that the increase in arc length around the 13th second is significant. In this moment, the welder had lifted the rod tip and as a result the arc length was so long that it neatly caused the arc’s extinction. Moreover, problems with arc ignition are also visible at the welding procedure’s beginning, where the mean feature value is 0 (same the standard deviation). A similar situation can be found when analyzing the DC(−) polarity (Figure 19a). The standard deviation $\sigma_{V\_DIAG}$ signal values were also elevated when welding with a long arc. It is generally lower for the negative polarity, which is caused by the smoothing in feature calculation, because high impulses, provoked by arc instability, are in this case removed. Applying the welding arc length indicator ($z_{ALI}$) with the limits mentioned in Section 3.2., a decisive signal can be extracted. When dealing with raw $z_{ALI}$ values, in each procedure a smaller or larger number of regions suspected of exceeding the optimal arc length can be found.

**Figure 20:**
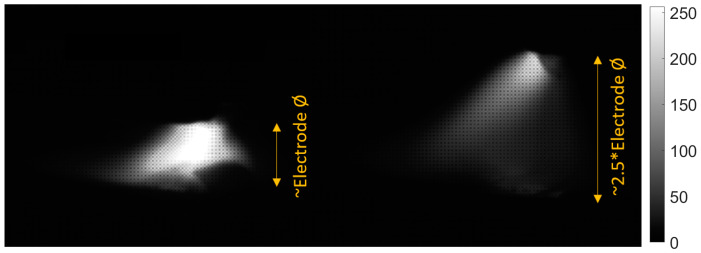
Arc length luminance and geometry variation, DC(+), 150A, at the begin of the arc glowing (left) and before arc extinction (right).

In the trails made on the arc length test stand, for the DC(+) process carried out with current set on 150 A, the maximal arc length was equal to about 250% of the electrode diameter (Figure 20). The same ratio can be observed in the feature presented in Figure 13, Figure 14 and Figure 15a. The stable arc glows when the feature is below the 10 mm level, while for the long arc, the value tends to 40 mm. For the negative polarity, arc elongation can be even higher in terms of deviation from the optimal arc length (more than 300%). This makes it possible to calibrate the VIS CAM measurement to track arc length in the dimension units.

Assessing the polarity, to avoid application of negative polarity, in which the heat is mostly transferred to the electrode tip, not in the joint direction, is an important task, especially during MMA training. It was found that for the negative polarity, the span between the minima of the diagnostic signal is expressed as $\min\left(x_{V\_DIAG2}\right)={\bar{x}}_{V\_DIAG2}-\sigma_{V\_DIAG2}$, and the multiplicity of standard deviation is larger, as for the positive polarity. While the diagnostic signal is calculated as a product of ellipse eccentricity and ellipse minor axis length (the ellipse that has the same second order moments as the arc ROI), it is easy to explain this phenomenon. For positive polarity for the same distance between electrode tip and work, the arc cone is quite narrow, and thus it has low eccentricity, while the corresponding ellipse is more elongated than circular and at the same time the minor axis length of the ellipse is small. For the negative polarity, the arc moves round the electrode tip. In the VIS CAM image, due to a relatively long exposition time, the arc cone diameter is apparently wider. Thus, the eccentricity is higher, as in the case of DC(+) polarity. At the same time, due to blurry arc representation, the standard deviation is lower, because in a wide range, the arc moves within the apparent bright arc cone. Having a large mean value and small standard deviation of $x_{V\_DIAG\_2}$ signal is a key factor to assign polarity indicator. This relationship is clearest for the short arc case, where ${\bar{x}}_{V\_DIAG2}$ and $\sigma_{V\_DIAG2}$ are close to each other for the DC(+), being for the similar case separated when DC(−) is used.

According to the obtained arc length indicators that have been used to assess process stability and to polarity indicators describing the direction in which DC current is supplied to the arc, two quantitative metrices were proposed to judge the correctness of arc length and polarity type. The arc length index describes the ratio between the sum of the arc over length indicators $al_{i}$ in a certain time window and the length of the time windows expressed in a total number of recorded images in this time range $n_{\Delta ti}$:

(4) $$al=\frac{1}{n_{\Delta ti}}\sum_{i=1}^{\Delta ti}al_{i}$$

In a similar way, the polarity index was defined, where $pl_{i}$ is the number of polarity indicators, which point to the presence of negative polarity in a certain time moment:

(5) $$pl=\frac{1}{n_{\Delta ti}}\sum_{i=1}^{\Delta ti}pl_{i}$$

The results obtained for all considered arc length classes and polarities are gathered in Table 3. Indexes al and pl were calculated for the whole realization of the process and parts of the process. When realizations of parts of the process were undertaken, the whole signal was divided into three even fragments. In the first, the influence of arc ignition is present, and the second one should be the most stable part of the process, while the last one contains the arc extinction. After investigation of the results, it was revealed that there is a clear border between the short and long arc and between positive and negative polarity.

The arc length index calculated for the whole signal length is below 0.1 for the short arc (the stable process with DC(+) polarity), below 0.15 for DC(−) polarity with a short, stable arc, and above 0.80 for a long arc that is associated with an unstable process. When looking at a shorter fragment of the process, the al increases, and the rise is more significant for the neutral polarity. In addition, observing the change in the al index, it is possible to point out when the arc has crossed the limit level, and the process is thus going to be unstable. According to that, it is possible both to inform a welding trainee that the distance between the electrode tip and the welded material is too long or to signal to an experienced welder or welding trainer that the process has become unstable.

A similar situation is seen with the polarity index pl. For the short arc, there is a considerable gap between pl values expressing positive and negative polarity. For the long arc, the values are high for both polarities, making the distinction among them impossible. This is caused because the index is vulnerable to large fluctuations in the arc shape in the start and extinction phase of the MMA process conducted with a long arc. When only the middle part of the process is quantified, the interval between polarities is much higher, because the value for the positive polarity average is ${\bar{pl}}_{DC\left(+\right)}$ = 0.14, while for negative polarity it is ${\bar{pl}}_{DC\left(-\right)}$ = 0.34.

## 4. Prototype of End-User Device

**Figure 21:**
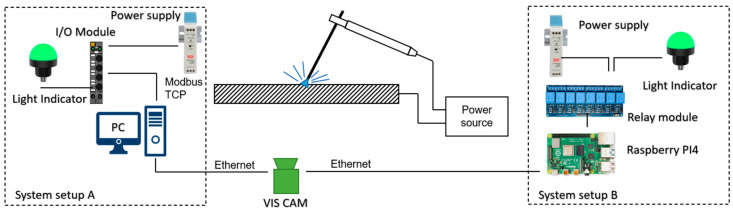
End-user weld monitoring systems setups, with the PC as a central unit (setup A) and a low-cost portable design with Raspberry Pi4 (setup B).

**Figure 22:**
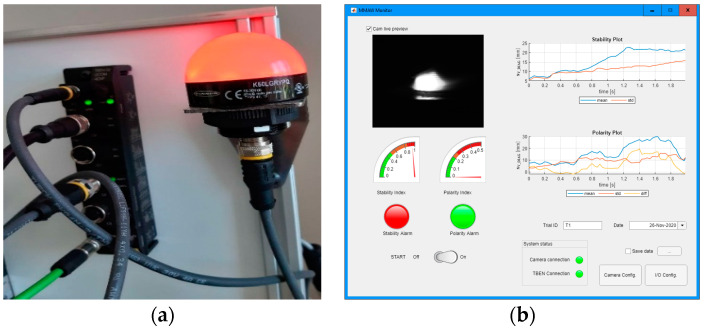
(**a**) View of I/O module—Turck TBEN TBEN-S2-2COM-4DXP, Turck GmbH & Co. KG, Mulheim, Germany, and indicating light Banner K50LGRYPQ; (**b**) screenshot of System A GUI, made in Matlab App Designer.

To allow further testing of the proposed method and to ease the method application in welding training, a prototype of a stand-alone welding monitor device was integrated. There were two types of device proposed, differing in the control unit hardware and the availability of a GUI (Graphic User Interface, Figure 21). In the first version, the system control unit was a PC, with Matlab 2020b application that offered a GUI for the user (Figure 22b). In the second proposition, the system was made more portable. The control unit was a Raspberry Pi4 B (RP4), and in this case, there was no GUI for the user. The RP4 computer was selected, because previous versions of Raspberry Pi were not equipped with a full Gigabit Ethernet (this was emulated by USB2). Because of the relatively low processing capability of the RP4, the image acquisition was made with a reduced frame size (half of the original image). This does not influence the method selectivity, but in this case, processing was made only at maximal 5 fps. The application on RP4 was configured in offline mode, using an external monitor and configuration setup that was stored in .xml files. For both system setups, the communication with the welder and trainer is made through a light indicator (Figure 22a). Communication between the central unit and the LED indicator is made through an I/O block offering Modbus TCP in the case of setup A with PC, and via a relay module in the case of Setup B.

Additionally, in the Matlab GUI, an option to introduce a custom user camera and to change the working distance setting in order to properly apply the stability and polarity detection principle. Moreover, this setting preserves the comparability of results using various CMOS cameras, equipped with lenses characterizing by custom focal length.

The hardware used to build the monitoring system in setup B, where the processing is performed by a Raspberry Pi4 computer, makes the system an economically optimized alternative to commercial systems in the area of process stability assessment. Additionally, the used camera, in terms of offered resolution and acquisition speed, is on a similar level to off-the-shelf systems, where 30 fps is the currently offered level [40,41,42] (max. 55 fps [43]) and the spatial resolution is mostly around full HD level [40,41,42,43].

## 5. Conclusions

In this paper, an approach for assessing welding arc stability and polarity in MMA welding is presented. To evaluate both properties, a visible light camera was used. The proposed method has been confronted with arc voltage and infrared thermograms to confirm its stability. It has been found that joint application of geometry features applied for ROI containing binary representation of the welding arc can act as a diagnostic signal, which carries information about process stability. Appling a sliding window algorithm to calculate the average and standard deviation of the diagnostic signal allowed formulation of criteria that can be used to distinguish short (stable) and long unstable arcs, as well as the polarity type. Because for the proposed arc length and polarity index, the span between arc length and polarity classes is large, the method is a robust one, giving a marginal chance for misclassification (for the proven set of welded samples). It is a novel approach, because in the literature there are no other propositions on how to assess the MMA polarity with the use of a visible light vision system. The proposed approach can be used to aid MMA welder training. In addition, it can be introduced to monitor linear butt welding in harsh environments, e.g., in underwater welding, where the welder is deprived of the possibility of using their hearing for evaluation of process stability and their sight is also providing limited information.

Monitoring of welding processes is a vital issue, and many research teams and industrial companies are working on proposing a robust and reliable tool for the quantification of welding processes and the resulting joint quality. The majority of efforts relate to automatized or robotized methods, where process parameters are key factors of process stability and they determine the weld quality. In MMA, keeping the desired parameters is mainly in hands of the welder, because he must maintain a constant arc length while moving the electrode tip toward the welded material and moving the electrode across the welding groove. This demands a certain skill. The proposed approach is an innovative one, because it gives a feedback to the welder (during the training) when he keeps the arc at constant length, and when the arc length has changed, and it is too long. This is a novel solution, different from those in which high frequency measurements of welding parameters are demanded. It is also other type of educational aid, other than virtual welding devices. As the obtained results show, this method is a good start for future development, while in its current form, it provides valuable information to the welder, with a time delay of 1/30 s.

The aim of the research was to evaluate the stability of the manual welding process with coated electrodes based on the analysis of the arc length (which reflects arc voltage). The obtained results make it possible to control the process in terms of maintaining the appropriate arc length, which translates into the correctness of the welding process and the elimination of welding incompatibilities.

In summary, the most important conclusions are:

- The proposed approach is suitable for the detection of too long a welding arc and distinction between a positive and negative polarity of welding current,
- Using visible light cameras, the user response can be made in an online mode, that is only limited by camera speed (30 fps),
- A prototype was elaborated, that can be easily applied in real-life applications and tuned to user camera. Additionally, it is robust to changes in the stand’s geometrical setup.

To generalize the proposed method, it is necessary to perform further studies on a larger set of joints that must be made with various parameters. Because in the current stage the proposed approach was only assessed for down-hand welding (cladding), its validity to other types of welding, e.g., overhead welding, will be investigated in the future research. The next step should also be the application of system other than the Raspberry Pi4 computer. Implementation of the proposed algorithm on a computer with a strong GPU, such as NVIDIA Jetson or NVIDA Xavier, could be an option to increase the performance.

## Figures and Tables

**Table 1:** Chemical composition in % of weight and typical mechanical properties of BASIC 7018 electrodes.

C	Mn	Si	HDM
0.05%	1.3%	0.4%	4 mL/100 g
**Yield strength [N/mm^2^]**	**Tensile strength [N/mm^2^]**	**Elongation [%]**	**Impact ISO-V [J]**
475	540	27	105 (at −40 °C)

**Table 2:** Chemical composition in % of weight and typical mechanical properties of S355J2 steel.

C	Mn	Si	P		S	Cr	Ni		Mo	W		V	Al	Cu
0.2	1.5	0.2–0.5	≤0.04		≤0.04	≤0.3	≤0.3		-	-		-	≤0.02	≤0.03
**Yield strength [N/mm^2^]**				**Tensile strength [N/mm^2^]**				**Elongation [%]**			**Hardness [HB]**			
>355				490				>25			230			

**Table 3:** Arc length and polarity index values calculated for raw arc length indicator and polarity indicator vectors.

Welding Scenario	Arc Length Index al Whole	Arc Length Index al Begin/Middle	Process Polarity pl Index Whole	Process Polarity Index pl Middle
DC(+) stable	0.01	0.00/0.01	0.10	0.14
DC(+) unstable	**0.84**	**0.80/0.84**	**0.29**	0.14
DC(+) mixed	0.51	0.00/0.56	0.14	**0.15**
DC(−) stable	0.06	0.12/0.00	0.26	0.36
DC(−) unstable	**0.97**	**0.99/0.94**	**0.28**	**0.31**
DC(−) mixed	0.45	0.13/0.22	0.23	0.35

## Data Availability

The data presented in this study are available on request from the corresponding author.
